# A Scoping Review of the Use of Artificial Intelligence in the Identification and Diagnosis of Atrial Fibrillation

**DOI:** 10.3390/jpm14111069

**Published:** 2024-10-24

**Authors:** Antônio da Silva Menezes Junior, Ana Lívia Félix e Silva, Louisiany Raíssa Félix e Silva, Khissya Beatryz Alves de Lima, Henrique Lima de Oliveira

**Affiliations:** 1 Faculty of Medicine, Federal University of Goiás, Goiania 74690-900, Brazil; ana.felix@discente.ufg.br (A.L.F.e.S.); khissya_beatryz@discente.ufg.br (K.B.A.d.L.); henrique.lima2@discente.ufg.br (H.L.d.O.); 2 Faculty of Medicine, Pontifical Catholic University of Goiás, Goiania 74605-010, Brazil; 3 Faculty of Medicine, University of São Paulo, São Paulo 01246-903, Brazil; louisiany.silva@cs.unicid.edu.br

**Keywords:** artificial intelligence, atrial fibrillation, diagnosis, scoping review

## Abstract

Background/Objective: Atrial fibrillation [AF] is the most common arrhythmia encountered in clinical practice and significantly increases the risk of stroke, peripheral embolism, and mortality. With the rapid advancement in artificial intelligence [AI] technologies, there is growing potential to enhance the tools used in AF detection and diagnosis. This scoping review aimed to synthesize the current knowledge on the application of AI, particularly machine learning [ML], in identifying and diagnosing AF in clinical settings. Methods: Following the PRISMA ScR guidelines, a comprehensive search was conducted using the MEDLINE, PubMed, SCOPUS, and EMBASE databases, targeting studies involving AI, cardiology, and diagnostic tools. Precisely 2635 articles were initially identified. After duplicate removal and detailed evaluation of titles, abstracts, and full texts, 30 studies were selected for review. Additional relevant studies were included to enrich the analysis. Results: AI models, especially ML-based models, are increasingly used to optimize AF diagnosis. Deep learning, a subset of ML, has demonstrated superior performance by automatically extracting features from large datasets without manual intervention. Self-learning algorithms have been trained using diverse data, such as signals from 12-lead and single-lead electrocardiograms, and photoplethysmography, providing accurate AF detection across various modalities. Conclusions: AI-based models, particularly those utilizing deep learning, offer faster and more accurate diagnostic capabilities than traditional methods with equal or superior reliability. Ongoing research is further enhancing these algorithms using larger datasets to improve AF detection and management in clinical practice. These advancements hold promise for significantly improving the early diagnosis and treatment of AF.

## 1. Introduction

Atrial fibrillation [AF] is the most frequent arrhythmia observed in medical practice and is characterized by disorganized and rapid electrical activity in the atria, leading to ineffective contractions. Over time, atrial tissues undergo structural changes, including fibrosis and damage to conductive tissues, contributing to the chronicity of AF [1,2]. Age is the primary risk factor for AF, with its incidence and prevalence increasing globally as life expectancy increases [3]. Other triggers include genetic predisposition, inflammation, and stress, as well as conditions, such as hypertension, heart failure, myocardial infarction, and diabetes [4,5,6,7]. Early identification of these risk factors is crucial for preventing disease progression [8,9].

AF affects approximately 37 million people worldwide, and its prevalence is expected to increase significantly [10]. Estimates suggest that by 2050, over 16 million Americans will develop AF, highlighting the urgent need to implement effective preventive strategies [11]. AF is associated with several severe outcomes, including increased mortality risk, cardiovascular events, stroke, heart failure, and kidney disease, with heart failure having the most significant absolute risk increase [12]. These complications underscore the importance of early and accurate diagnosis.

While 12-lead electrocardiography [ECG] remains the gold standard for diagnosing AF [13,14], it has limitations due to its low sensitivity, especially in detecting paroxysmal or asymptomatic cases [13,15,16]. The growing use of artificial intelligence [AI] in healthcare offers potential improvements for detecting and diagnosing AF. AI algorithms, particularly those that employ machine learning [ML], can analyze large datasets, identify patterns, and enhance diagnostic precision [17,18,19,20]. These advancements can refine AF detection and facilitate earlier treatment, mainly through techniques such as deep learning [DL], which uses neural networks to extract complex features from raw data [18,21].

This scoping review aimed to map the current applications of AI, particularly ML, in detecting and diagnosing AF in adults. This review categorizes and compares approaches by exploring various AI techniques and identifying trends and gaps to guide future research.

## 2. Materials and Methods

This review was conducted according to the Preferred Reporting Items for Systematic Reviews and Meta-Analyses Extension for Scoping Reviews [PRISMA-ScR] guidelines. Searches were carried out in databases such as PubMed, Excerpta Medica Database [EMBASE], and Scopus until April 2024, using MeSH terms related to AI and the diagnosis of AF. The bibliographic references of relevant articles were also included in the review.

### 2.1. Eligibility Criteria

This review focused on articles that addressed the identification and diagnosis of AF using AI, specifically ML techniques. We included studies published in peer-reviewed journals up to April 2024 that utilized AI models to improve the effectiveness of AF diagnosis. The scope of this review covered quantitative, qualitative, and mixed studies that validated, trained, and/or tested the feasibility of ML models, such as neural networks, for predicting and identifying AF by various means, such as electrocardiography, photoplethysmography sensor signals, imaging tests, and medical notes.

### 2.2. Exclusion Criteria

Secondary sources, such as editorials, books, expert opinion articles, dissertations, theses, and conference abstracts, were excluded from the review. Literature reviews relevant to this theme were included. Studies that did not use ML models were excluded.

### 2.3. Protocol and Registration

This scoping review was registered in Open Science Framework [DOI registry 10.17605/OSF.IO/9FYG3].

### 2.4. Data Sources

We extensively searched the EMBASE, SciVerse Scopus, and PubMed databases. The search strategy involved combining controlled descriptors and keywords pertinent to the use of AI to diagnose AF. No restrictions were imposed on the language or timing of the publication. Additionally, the reference lists of the initially selected studies were manually searched to identify other relevant articles.

### 2.5. Study Selection Process

The identified studies were imported into Rayyan software (Version 2023) to remove duplicates and evaluate the eligibility criteria [12]. Two independent blinded reviewers first screened titles and abstracts [phase 1] and then reviewed the full texts of the studies selected in phase 1 [phase 2]. Any discrepancies during the selection process were resolved by the reviewers.

### 2.6. Data Extraction Process

Two independent blinded reviewers extracted the data using a characterization table created in Microsoft Word (2021). The table included study characteristics such as author, study design, year, and country; main results such as the improvement in the sensitivity of the diagnosis of AF with the use of AI; and the study conclusions.

### 2.7. Data Synthesis

Data from the selected studies were qualitatively synthesized, focusing on the use of ML as a model for predicting and identifying AF. The synthesis included an analysis of AI techniques to improve AF prediction, identification, and diagnosis algorithms. All information was organized into a descriptive table for comprehensive analysis.

## 3. Results

**Figure 1:**
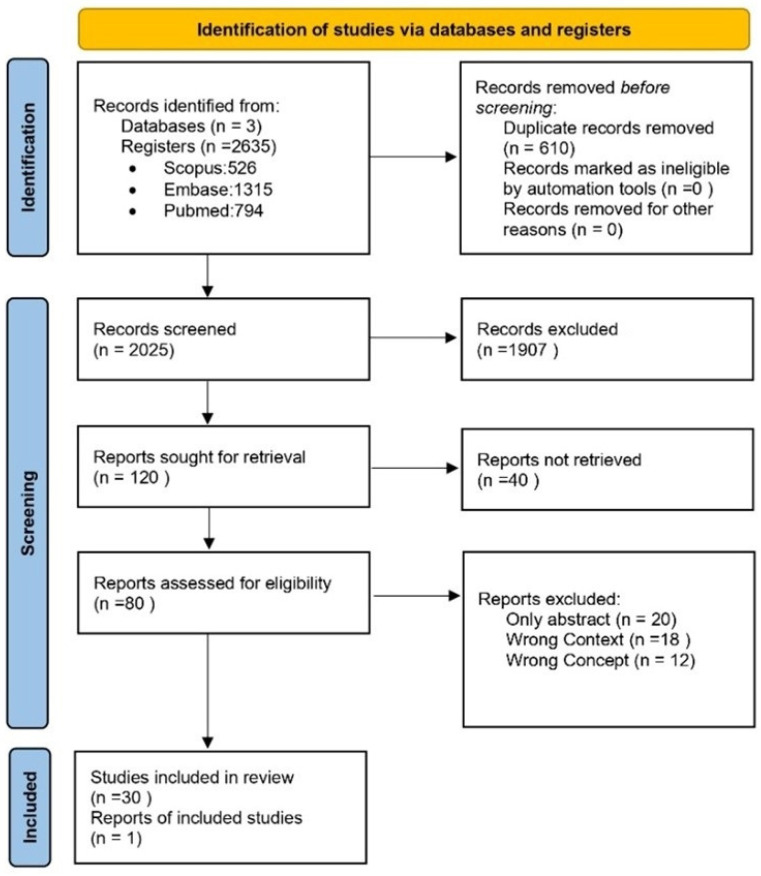
PRISMA flowchart.

In total, 31 articles were selected for the final analysis from an initial search of 2025 articles [Figure 1]. The primary data from these studies are presented in Table 1. The analysis focused on three main categories: [1] development of an ML algorithm for AF detection, [2] comparison of methods and algorithms [3], and development of predictive models for AF detection.

### 3.1. Development of ML Models for AF Detection

Given the need to optimize the diagnosis of atrial fibrillation [AF], several studies have been conducted to create and validate machine learning [ML] algorithms capable of detecting AF from electrocardiogram [ECG] signals, photoplethysmography [PPG], medical records, and imaging exams. The goal is to verify the selected method’s sensitivity, specificity, area under the ROC curve, and positive and negative predictive values [39,40].

The methodology used to develop ML-based algorithms requires a large dataset to train and test the discriminatory ability of the method. Cross-validation, for example, is used to improve the model’s performance and prevent it from making incorrect inferences when exposed to unknown populations. This involves randomly dividing the data into equal-sized subsets, each containing a percentage of values for training and another for validation, which are processed sequentially by the algorithm until a complete reading of the data is achieved to assess the algorithm’s effectiveness. In addition to this technique, the model can be applied to an external population to ensure good performance [20].

Data extraction is a key step in developing and validating machine learning models. The main sources of information used by the studies included in this review were hospital records, public repositories, complementary exams, or wearable devices like smartwatches, followed by noise and artifact removal. The sample was then divided into training, validation, and testing sets, with the latter kept separate until the algorithm was ready for evaluation to ensure an unbiased analysis of its performance. For instance, one study divided the patient cohort into 70/10/20, with 70% of the data used for training the network, 10% for internal validation, and 20% for testing the model. This study used samples of AF and sinus rhythm [SR] from simulated ECGs and then explored a large ECG database with known rhythm status to train the ML algorithm, a fundamental step in machine learning [22].

The most frequently used model in the selected studies was the convolutional neural network [CNN], as it is better at identifying multidimensional relationships in nonlinear domains than traditional or linear regression approaches, resulting in greater efficiency when analyzing large data volumes. The Alerte AI platform, for example, can identify AF from any continuous ECG monitoring device, allowing feedback and continuous algorithm training [18,22]. In addition to CNNs, some studies used supervised algorithms, such as support vector machines [SVMs], random forests, and gradient-boosting machines [GBMs], to distinguish between normal heartbeats and those indicating AF. In some cases, reinforcement learning can be used, as it progressively improves accuracy by interacting with simulated environments and receiving performance feedback. Finally, validation and testing are conducted to ensure the model’s efficacy. Algorithms are evaluated based on metrics, such as sensitivity, specificity, accuracy, positive predictive value [PPV], negative predictive value [NPV], and the area under the ROC curve [AUC-ROC]. The graph shows the relationship between the true-positive rate [sensitivity] and the false-positive rate [18,22].

In China, researchers developed a network architecture called DenseNet-BLSTM for automatic AF detection using ECG signals. DenseNet is a convolutional neural network characterized by dense connections between its layers, where each layer receives outputs from all previous layers. This configuration ensures the efficient reuse of features and improves gradient flow, resulting in faster and more effective training. The DenseNet-BLSTM integrates the DenseNet architecture, which captures local data characteristics, with a bidirectional long short-term memory [BLSTM] model, which captures long-term temporal dependencies, crucial for analyzing time-series data such as ECG. Thus, this combination allows for the extraction of both spatial and temporal features from the evaluated data. The model achieved an accuracy of 99.07% in the training set, 98.15% in the validation set, and 97.78% in the test set, demonstrating high reliability in the automatic detection of AF [36].

A different approach is the use of transfer learning, in which a network previously trained on a large dataset is adapted to perform a new task on another dataset without requiring new training. The feasibility of this method for AF detection was examined in a pioneering study conducted in Australia, in which single-lead ECG traces recorded during laboratory sleep studies were selected for analysis. This model detected all AF cases, demonstrating excellent negative predictive value, although specificity and overall diagnostic accuracy were below 80% [26].

A deep neural network model was developed to detect small variations in 12-lead ECGs during normal sinus rhythm that indicated the presence of paroxysmal AF [PAF]. The researchers hypothesized that the area around the P-wave preceding the QRS complex would be crucial for identifying AF. Thus, the model was tested with different time interval sizes before the QRS complex until it was determined that the optimal interval for identifying subtle changes indicating PAF is approximately 0.24 s. This interval, identified as a critical section for differentiating PAF from normal sinus rhythm, could be used in the future to select high-risk patients who would benefit from better investigation of this arrhythmia [13].

In the context of attempting to detect paroxysmal AF during sinus rhythm on ECG, researchers in South Korea combined a convolutional neural network [CNN] and a gradient-boosting machine [GBM]. The sample consisted of patients who underwent 24 h Holter monitoring, excluding those who exhibited documented AF or pacemaker rhythm during the examination. The CNN was responsible for extracting and identifying relevant patterns in each 7 s segment of ECG, while the GBM was used to capture temporal information throughout the 24 h ECG recordings. Thus, the model achieved high efficacy in detecting paroxysmal AF, especially during nighttime, and sensitivity was higher as the time between the Holter test and the patient’s AF diagnosis decreased [41].

Li et al. developed a self-complementary attention convolutional neural network [SC-CNN] to accurately identify AF in dynamic wearable ECG signals. To better capture the characteristics of ECG signals, a “Z” reconstruction method was used to convert the 1D signal emitted by the device into a 2D matrix, allowing the convolutional network to explore information from both adjacent and more distant sample points over time. Feature extraction was performed by the Surface Feature Extraction [SFE] module, which consisted of three convolutional blocks. Subsequently, the Self-Complementary Attention Mechanism [SC-Net] refined the data and captured critical information from the ECG signals for AF identification. Applying the model to three public datasets resulted in AUC values of 99.79%, 95.51%, and 98.77%, indicating that the model is near ideal, as it significantly and reliably classifies samples as positive or negative. In clinical datasets, the model achieved a sensitivity of 99.62%, demonstrating its efficacy in identifying AF in real clinical scenarios [28].

In Finland, a team evaluated the feasibility and accuracy of the entire operational sequence of an arrhythmia monitoring system using an automated wearable device for AF detection. This mHealth solution included a consumer-level single-lead ECG chest belt [Suunto Movesense, Suunto, Vantaa, Finland], a mobile app, and a cloud service with an AI-based arrhythmia detection algorithm [Awario, Heart2Save]. A simultaneous 3-lead Holter ECG recording was taken as a gold-standard reference for rhythm classification. In this study, 80% of the records obtained with the chest belt were adequate for visual and automatic AF diagnosis, with 100% diagnostic accuracy. Additionally, the Awario algorithm demonstrated 100% sensitivity and 95.4% specificity [with four false positives] in detecting AF. Regarding user experience with the chest belt, using the device was preferred compared to performing a Holter ECG examination [25].

In addition to ECG-based methods for AF detection, some studies applied ML algorithms to photoplethysmography [PPG] recordings taken from the tip of the index finger using a smartphone camera with optical sensors [30,34,42]. One study selected 79 patients who had undergone cardiac surgery for heart rhythm monitoring using PPG signals collected by an Apple Watch [series 4]. PPG data features were extracted every minute, including the mean and standard deviation of heart rate over a 10 min interval before identifying AF episodes. The selected AI model, the Gradien-Boosting Decision Tree [GBDT], had an AUC of 0.9416, sensitivity of 90.9%, and specificity of 83.8%, indicating excellent discriminatory ability between patients with and without AF [34].

In a subsequent study, an AI algorithm achieved a specificity of 99.1% and sensitivity of 89.9% in distinguishing between sinus rhythm and AF using PPG signals captured by conventional smartphones. Thus, this method emerges as an accessible and practical option for AF screening due to the widespread availability of smartphones and the easy reproducibility of the assessment [30].

Given the numerous studies on the development of machine learning algorithms for AF identification, there arises a need for a better understanding of the steps undertaken by the algorithm and greater clarity regarding the interpretation of results. To this end, a group of researchers in Japan developed an ECG interpretation model and applied the Gradient-weighted Class Activation Mapping [Grad-CAM] method. This technique is used in convolutional neural networks to visualize and interpret which regions of an image were most relevant to the model’s decision, thereby making the diagnostic process more transparent and understandable [23].

Similarly, a group in Spain applied explainable artificial intelligence [XAI] techniques to a set of images called PhotoplethysmoMatrix [PPM], derived from PPG signals classified into sinus and AF rhythms by neural networks. XAI was implemented as an additional layer to the CNN, which adjusts the model’s attention to both spatial aspects and image channels. The aim is to better identify the areas of the sample that are most important for distinguishing between AF and sinus rhythm. Therefore, as discussed in the studies above, this subfield of AI aims to create models that enable humans to understand how and why AI reached a certain conclusion, thereby strengthening user confidence in the system [33].

Furthermore, creating algorithms to detect AF is not the only important factor; models that improve detection through data expansion are also essential. In this context, an incremental learning system with a loop-lock structure combining transfer and active learning was developed [43]. The initial stage of the process involves pre-training the Multiple-Input Deep Neural Network [MIDNN] model using labeled samples from large databases, such as the MIT-BIH Atrial Fibrillation Database [AFDB]. During practical application, the algorithm continuously collects unlabeled ECG rhythm data. The active learning strategy is then applied to select the most informative samples, considering the uncertainty of predictions and RR interval variability. Subsequently, the selected samples are sent to experts for labeling, and the model is refined with these new data. This cycle repeats progressively, and with each iteration, the system’s accuracy increases, even with minimal manual intervention. This research reported, after 14 iterations, accuracy, sensitivity, and PPV indices of 97.53%, 100%, and 95.29%, respectively, in detecting AF, demonstrating the efficacy of the proposed model. Moreover, the active learning strategy achieved this improvement with more than a 90% reduction in labeling costs [43].

Another study evaluated whether adding a two-step AI filter could increase the positive predictive value [PPV] of AF episodes detected by an implantable loop recorder [ILR]. This filter consists of a deep neural network with two components: one that analyzes the entire ECG signal and identifies QRS complexes, and another that predicts the presence or absence of AF in 10 s windows by analyzing the period preceding the QRS complex to detect P-waves and exclude false positives [FP]. The study observed an improvement in the PPV of AF episodes detected by the ILR, from 53.9% to 74.5%, with the FP rate reduced by up to 66%. Therefore, integrating AI algorithms into cardiac monitoring devices can significantly improve arrhythmia detection accuracy, reduce clinical workload, and improve patient outcomes [37].

Innovatively, Shah et al. developed a portable tool and a natural language processing [NLP] algorithm to identify AF patients through clinical notes extracted from electronic health records, which included information on medical history, test results, and symptom descriptions. The data were separated into training, internal validation, and external validation. The proposed model identified AF patients with 92.5% sensitivity and 88.7% specificity. Although its efficacy decreased when applied to data from an external healthcare system, the results remain promising for using this tool in clinical practice [44].

Similarly, an NLP algorithm was developed and applied in the USA to identify recurrent episodes of AF after the initiation of antiarrhythmic therapy using electronic medical records. Compared to an approach based solely on codes, it was more sensitive in identifying patients with recurrent AF, making it highly useful for large population analyses [27].

### 3.2. Comparison of Methods and Algorithms

Traditional AF screening methods include 12-lead ECG, dynamic ECG, pulse palpation, and implantable ECG monitoring. Although conventional ECG is used to diagnose paroxysmal AF, it may fail to detect short-duration episodes and is only performed in hospital settings. Dynamic ECG, on the other hand, extends the monitoring period and increases the detection rate of paroxysmal AF. However, prolonged monitoring may interfere with patients’ daily activities and cause skin irritation. Pulse palpation can detect abnormalities and improve paroxysmal AF detection, but it is a difficult technique to learn and reproduce. While offering high sensitivity and specificity in AF diagnosis, implantable ECG monitoring equipment is expensive, invasive, and not feasible for large-scale use. In this context, low-cost wearable electronic devices have great potential for promoting long-term rhythm tracking and monitoring [45].

In Turkey, a clinical study evaluated the efficacy of a convolutional neural network [CNN] with DenseNet architecture, called CurAlive, in classifying AF rhythms, sinus rhythms, or other arrhythmias using only data extracted from a single-lead ECG [ECG-I]. CurAlive is a cardiac monitoring technology that includes two ECG devices [one with a single lead and one with six leads], an AI-managed ECG data analysis system, and integrated PPG sensors to measure oxygen saturation and body temperature. The data collected by the devices are processed by a CNN trained on large databases, such as PTB-XL, which contains a wide range of ECG signals, MIT-BIH, specialized in AF, and a noise-specific database of the CurAlive technology itself.

To verify system accuracy, the model’s performance was compared to the evaluation by three independent cardiologists regarding interpreting 10 to 30 s ECG-I recordings, classifying them as normal sinus rhythm, AF, others, and noise. Subsequently, the performance of both groups was compared to the corresponding classification of 12-lead ECGs. The AI model achieved an overall accuracy of 93.6%, demonstrating high selectivity for all groups, while the average accuracy of the specialists was 54.6%. Regarding AF detection, AI exhibited excellent performance, achieving 100% precision and an F1 score of 98%. Therefore, these results suggest that CurAlive can be a useful tool for cardiologists to quickly and effectively monitor patients with ECG abnormalities, especially in telemedicine contexts [46].

Faced with the challenge of detecting AF due to its paroxysmal nature, inexpensive devices capable of capturing long-term data for AF screening stand out as desirable alternatives to traditional screening methods. Recent studies have demonstrated that PPG-based applications can differentiate between AF and sinus rhythm with sensitivity and specificity greater than 90%, and that the AF detection rate is three-times higher when using internet-enabled single-lead mobile ECGs [iECGs] connected to smartphones compared to routine monitoring [38,47].

Considering this, a clinical validation study conducted in Switzerland and Germany [30] selected 592 patients for heart rhythm analysis using PPG and mobile ECG recording. The aim was to validate the performance of an automated PPG-based algorithm in differentiating AF and sinus rhythm [SR] and to compare its results with the interpretation of an iECG by two blinded cardiologists. Patients underwent two stages of monitoring: [1] placing the right and left index and middle fingers on the corresponding electrodes of an iECG [AliveCor^®^, Mountain View, CA, USA] for 1 min, and [2] placing the index fingertip on the camera of a standard smartphone [iPhone 4S, Apple^®^], using a study version of the Heartbeats app [Preventicus^®^, Jena, Germany] for pulse wave recording for 5 min. The Heartbeats algorithm [version 20171120] analyzed the PPG signals, which discriminate between SR and AF based on pulse wave time and morphology analysis, as described elsewhere [48].

Two blinded cardiologists analyzed the internet-enabled ECGs, with an additional third blinded expert in cases of diagnostic uncertainty. Another algorithm, the Kardia Monitor [version 4.2.0.1487], which can automatically classify iECG data as “possible AF”, “normal”, or “unclassified” ,was validated against cardiologist diagnoses in additional analysis. Thus, 1, 3, and 5 min PPG records were analyzed by Heartbeats, and 1 min iECG records of the same patients were analyzed by specialists and the Kardia Monitor algorithm. Although the current standard for AF screening is a Holter ECG device, the Heartbeats algorithm achieved a specificity of 99.1% and a sensitivity of 89.9% in continuous 1 min PPG segments. Extending the analysis time from 1 to 5 min did not significantly improve test results and caused a considerable reduction in the signal quality of processed files. The Kardia Monitor algorithm achieved a specificity of 99.6% and a sensitivity of 97.8% in the files deemed suitable for automated interpretation.

Notably, both rhythm monitoring methods are effective in detecting AF. However, the technique using PPG signals offers the advantage of being applied directly through smartphones, eliminating the need for extra devices, making its use more accessible, practical, and promising [30].

To optimize AF identification, the performance of a wearable domestic ECG recorder was compared with a 12-lead ECG in different postures and after physical activity to simulate daily life. The wearable ECG data were transmitted to the Amazfit CardiDoc app and interpreted by the SEResNet neural network. In the supine position, the diagnostic consistency compared to the 12-lead ECG was 94.74%. In comparison, in the upright position, it was 97.37%, with similar results observed after exercise, confirming the device’s reliability in detecting AF in different positions and circumstances [45].

Another study validated the accuracy of a smart wristband device [Amazfit Health Band IS] that uses a combination of PPG and single-lead ECG systems, along with an AI algorithm [SEResNet], for AF detection. PPG signals were collected for 71 s, and if the algorithm detected a suspected AF, the collection was repeated to check for recurrence. The ECG exam required the user to touch an external electrode to start a 60 s recording. The sensitivity, specificity, and accuracy for AF detection using PPG were 88%, 96.41%, and 93.27%, respectively, while for ECG, they were 87.33%, 99.20%, and 94.76%. The integration of these technologies provided reliable and advantageous results for AF detection, as PPG provides screening and low-energy consumption, while single-lead ECG provides recordings for medical review. Additional studies are needed to assess the device’s effectiveness in real-life environments, as this research collected data while patients were at rest [14].

A differentiated study was conducted with AF patients admitted for cardioversion, assessing the accuracy of adding an autonomous algorithm [Fibricheck] for reading PPG signals in a standard wristband and smartwatch to distinguish between AF and sinus rhythm. The Fibricheck algorithm was developed based on deep learning and can analyze PPG data to detect AF. The Biostrap [wristband] and the Fitbit Ionic [smartwatch] were used. The algorithm’s results were compared with 12-lead ECG analysis by medical experts. Both devices achieved sensitivity, specificity, PPV, NPV, and accuracy equal to or greater than 95%. The study demonstrates the feasibility of associating a machine learning algorithm with a wristband/smartwatch without a previously incorporated algorithm, facilitating the population’s access to AF screening processes [29].

Finally, with the same goal of evaluating the signal quality of a portable single-lead ECG device, a large-scale screening study in Germany developed a machine learning algorithm. The results indicated a correlation of 0.6 during tests compared to expert evaluations. This demonstrated the algorithm’s effectiveness in signaling when to repeat measurements, suggesting additional human reviews, and reducing incorrect automated classifications, optimizing the AI-assisted AF identification process [31].

### 3.3. Development of Predictive Models for AF Detection

According to clinical guidelines, a cardiologist diagnoses AF when the arrhythmia is identified for 30 s or more on a 12-lead ECG. However, this method may fail to identify more than 50% of cases, particularly in paroxysmal forms, requiring other strategies, such as long-term Holter monitoring, which is difficult to implement on a large scale [35]. Given this, risk prediction algorithms for AF using machine learning have been developed to accelerate and facilitate screening potential undiagnosed AF carriers.

The PULsE-AI study conducted a randomized, multicenter clinical trial in six primary care clinics in England. It sought to determine the effectiveness of an AF risk prediction algorithm based on machine learning, combined with diagnostic tests [ECG and KardiaMobile], to identify undiagnosed AF. Participants were divided into two groups: an intervention group that used the algorithm to identify high-risk individuals for AF and offer diagnostic tests and a control group that received routine clinical care. The AF risk prediction algorithm was developed using machine learning techniques and electronic medical record data from a retrospective cohort of nearly 3 million patients. The model used age, sex, BMI, blood pressure, and cardiovascular comorbidities to generate an AF risk score. Those who had a high risk score according to the algorithm underwent diagnostic tests with 12-lead ECG and portable ECG. The results showed that those identified by AI had a higher probability of being diagnosed with AF, confirming the value of this tool for discriminating high-risk individuals who could benefit from diagnostic tests [49].

To predict incident AF in a high-risk population, a study explored different machine learning algorithms, such as XGBoost, random forest, and gradient-boosting decision tree, with XGBoost initially selected for training with 17 features extracted from PPG signals and later optimized by including new variables and adjusting hyperparameters. This model demonstrated a sensitivity of 81.9% and specificity of 96.6% in predicting AF up to 4 h in advance. Compared to continuous 72 h ECG monitoring, which is considered the gold standard for AF diagnosis, it obtained compatible results, suggesting significant potential for AF screening in both clinical and non-clinical settings [32].

Other studies developed and evaluated deep learning algorithms to predict paroxysmal AF in patients with a history of embolic stroke of undetermined source [ESUS], such as the ResNet architecture with convolutional neural networks. This model integrated demographic data, such as age, sex, weight, and height, with processed ECG data, achieving an AUC of 0.827 in external validation with ESUS patients. When combined with atrial ectopic burden and left atrial diameter, the model’s performance was improved, reaching an AUC of 0.906, showing a superior ability to identify paroxysmal AF. A potentially better prognosis is expected for patients using arrhythmia identification methods with high sensitivity and accuracy, as it allows for timely secondary intervention [32,50].

A study based on three years of medical information from the Taipei Medical University clinic database aimed to create a predictive model capable of identifying the risk of recent-onset AF in elderly patients within one year. The machine learning algorithms used included decision tree, support vector machine [SVM], logistic regression, and random forest, with the latter proving to be the most effective, with an area under the curve [AUC] of 0.74 and a specificity of 98.7%. Moreover, the combined use of diagnoses, medication prescriptions, and laboratory data resulted in better model performance. Thus, this method could be used as an effective clinical alternative for predicting AF incidence in older patients [51].

Facing the difficulty of detecting paroxysmal AF, a machine learning algorithm was designed to predict this rhythm from a single-lead ECG in sinus rhythm rather than a 12-lead ECG. The convolutional neural network was modified to accommodate the longer length of the 30 s ECG signals, formatted into seven blocks, each consisting of two one-dimensional [1D] convolutions with kernel size three and a squeeze-and-excite step and residual connections. The algorithm was fed with data from three prospective AF screening studies, STROKESTOP I, STROKESTOP II, and SAFER. The main finding of the current study is that an AI algorithm interpreting a single-lead ECG can predict AF from normal sinus rhythm with acceptable accuracy for a homogeneous cohort in terms of age [AUC 0.62]. Still, it substantially improves for a cohort with age diversity [AUC 0.80]. Therefore, in a screening program, this method could potentially identify individuals who may benefit from additional examinations for AF diagnosis [52].

In addition to efforts to optimize screening for incident AF, research has been conducted to predict the recurrence of this arrhythmia. In this context, a group of researchers from South Korea conducted a cohort study to test whether left atrial wall stress could be predicted by AI using noninvasive parameters. This is because long-term atrial stretch can cause atrial dilation, which is a possible cause of AF progression and atrial remodeling. Therefore, predicting atrial wall stress could identify patients at higher risk of AF recurrence after catheter ablation. However, the algorithm achieved moderate accuracy, with an AUC-ROC of only 0.734 [53].

The most widely used catheter ablation technique to treat AF is pulmonary vein isolation, as these veins are the primary source of ectopic impulses. However, foci outside these veins can also trigger AF, especially in cases of recurrence after ablation. Faced with this challenge, the ResNet34 neural network was selected as a pre-trained model to predict the location of triggers in regions outside the pulmonary veins using computed tomography [CT] image analysis of patients with paroxysmal AF before catheter ablation. The technique achieved a predictive accuracy of approximately 82.4% and enabled the mapping of trigger foci for elimination during ablation, thereby reducing the risk of arrhythmia recurrence [54].

## 4. Conclusions

The development of ML algorithms for detecting AF represents a significant advancement in cardiology with the potential to enhance the diagnostic accuracy and effectiveness of treatments. Developing these algorithms involves a rigorous process that includes collecting and pre-processing large volumes of data, followed by sophisticated modeling and validation techniques. Models such as CNN and other advanced architectures have demonstrated high accuracy and reliability in detecting AF, surpassing traditional methods in some respects. Additionally, incorporating XAI and transfer learning techniques has made these algorithms more effective, transparent, and adaptable to clinical contexts. However, the application of such models still faces challenges, such as the need for continuous validation in real-world settings and integration with established clinical practices. Therefore, the success of these approaches depends on continuous technological evolution and acceptance of these methods by the medical community to ensure that the diagnosis and treatment of AF are increasingly accurate, accessible, and personalized.

The application of AI in healthcare, particularly for AF detection and management, presents significant opportunities but also faces numerous challenges, such as data quality issues, privacy concerns, integration difficulties, bias, and a lack of model interpretability. To address these, future research should focus on developing generalizable models, mitigating biases, enhancing model transparency, and fostering effective human–AI collaboration. Prospective clinical trials and research into the scalability and cost-effectiveness of AI solutions are necessary to validate their real-world impact. Addressing ethical and legal implications, improving wearable monitoring technology, and advancing personalized medicine are crucial for building trust and facilitating widespread adoption of AI in healthcare, ultimately enhancing patient outcomes and system efficiency.

## Figures and Tables

**Table 1:** Main characteristics of selected studies.

Authors	Country	Study Type	Main Objective	Main Results	Conclusions
Playford et al. [22]	Australia	Prospective validation study	Continuous electrocardiogram [ECG] recording using a wearable device with data transmission and artificial intelligence [AI] for rhythm monitoring.	Achieved 100% accuracy in classifying AF and sinus rhythm [SR] with ECG data [historical and simulated].	The AI model accurately classified AF and SR, demonstrating the potential of AI in diagnosing other arrhythmias.
Chen et al. [14]	China	Prospective cohort validation	Evaluate the sensitivity, specificity, and accuracy of a smart wristband equipped with photoplethysmography [PPG] and ECG systems to detect AF using AI.	PPG readings: Sensitivity 88.00%, Specificity 96.41%, Accuracy 93.27%; ECG readings: Sensitivity 87.33%, Specificity 99.20%, Accuracy 94.76%.	Smart wristbands using AI can reliably detect AF and SR, offering high accuracy in arrhythmia detection.
Fuster-Barceló et al. [23]	Spain	Model validation study	Develop and validate a methodology to classify AF using PPG signals converted into images.	Achieved 100% accuracy in classifying AF and normal sinus rhythm [NSR] using convolutional neural networks [CNN] and Explainable AI [XAI].	The methodology improved AF classification accuracy, ensuring transparency in the classification process with XAI techniques.
Liu et al. [24]	Taiwan	Algorithm validation study	Predict non-pulmonary vein triggers [NPV] before catheter ablation using deep learning models and image analysis.	Correctly predicted NPV triggers in approximately 82% of cases, with 64% sensitivity and 88% specificity. Overall accuracy improved to 89%.	Deep learning models can accurately predict NPV triggers, aiding decision-making before ablation procedures.
Santala et al. [25]	Finland	Diagnostic accuracy study	Evaluate the diagnostic accuracy of an automated mHealth system with wearable ECG, mobile app, and cloud service for AF detection.	Sensitivity: 100%, Specificity: 96%, and area under the curve [AUC] of 0.88, showing high predictive capacity.	The system provided high-quality ECG recordings and diagnostic accuracy, enhancing patient care.
Gahungu et al. [26]	USA	Retrospective validation study	Assess AI performance using single-lead ECGs recorded during polysomnography to detect AF.	AI achieved 100% sensitivity and 76% specificity.	AI application in sleep studies proved effective for AF detection, showing significant potential for integrated monitoring tools.
Korucuk et al. [27]	Turkey	Comparative analysis	Compare the AI-based AF detection model [“CurAlive”] with human interpretation of ECG data.	AI model achieved an accuracy of 94.1%, outperforming human interpretation [54.6%].	AI performed better than human experts, indicating its utility in supporting clinical decision-making.
Li et al. [28]	China	Algorithm development and validation	Develop and validate a convolutional neural network [CNN] for AF prediction from ECG signals.	CNN achieved 99.79% AUC, showing high robustness for real-time AF identification.	CNNs have strong potential for real-time, highly accurate AF prediction, particularly in wearable technology.
Guo et al. [29]	UK	Predictive model validation study	Develop an AI-based algorithm to predict AF onset 4 h before an event using patient data.	The model achieved an AUC of 94%, successfully predicting AF onset hours in advance.	AI’s predictive capacity enables pre-emptive diagnosis and decision-making, reducing AF complications.
Brasier, et al. [30]	Germany	mHealth validation study	Test accuracy of a smartphone-based PPG algorithm for AF detection compared to standard ECGs.	Achieved 99.1% specificity and 89.9% sensitivity.	The smartphone-based AF detection method offers a noninvasive, cost-effective solution for long-term monitoring.
Jeon et al. [31]	South Korea	Model development and validation	Developing an AI model to predict AF using various clinical markers, including ECG and demographic data.	The model demonstrated an accuracy of 92%, showing significant improvement over traditional AF detection methods.	AI models can be integrated into clinical practice to enhance AF prediction and early diagnosis.
Hygrell et al. [32]	Sweden	Prospective cohort study	Investigating AF detection using wearable ECG devices combined with machine learning algorithms for long-term monitoring.	Sensitivity: 94%, Specificity: 91%, significantly reducing AF detection time.	Wearable devices equipped with AI can improve long-term AF detection and reduce the time needed for diagnosis.
Shi et al. [33]	China	Retrospective validation study	Evaluation of AI performance using 12-lead ECGs for early AF detection in a large-scale clinical population.	AI demonstrated a sensitivity of 93% and specificity of 95% in detecting early-stage AF.	AI can enhance the early diagnosis of AF, particularly in asymptomatic patients.
Sánchez et al. [18]	Spain	Algorithm development and validation	Development of a deep learning algorithm for predicting AF recurrence post-ablation therapy.	The model achieved 85% accuracy in predicting AF recurrence, significantly improving treatment outcomes.	Deep learning models can support clinicians in predicting AF recurrence and tailoring post-ablation therapy.
Hiraoka et al. [34]	Japan	Predictive model development	Development of an AI algorithm to predict post-operative AF in patients undergoing cardiac surgery.	The model predicted post-operative AF with 87% sensitivity and 83% specificity.	AI models can aid in identifying high-risk patients and reducing post-operative AF complications.
Choi et al. [35]	South Korea	mHealth validation study	Validation of a PPG-based algorithm for AF detection using smartphone cameras.	Achieved 97.6% accuracy, 89.3% sensitivity, and 95.7% specificity.	Smartphone-based AI algorithms offer an accessible, low-cost solution for AF detection.
Tao et al. [36]	China	Algorithm development and validation	Development of an AI algorithm for detecting AF in patients with heart failure using ECG and other clinical markers.	The algorithm demonstrated 93% accuracy in detecting AF in patients with heart failure, outperforming traditional diagnostic methods.	AI algorithms can improve the early detection of AF in high-risk populations, particularly heart failure patients.
Zheng et al. [37]	China	Comparative analysis	Compare the performance of different AI models in detecting AF using ECG data from wearable devices.	The CNN-based model achieved the highest accuracy at 95.8%, significantly outperforming other machine-learning models.	CNNs offer superior accuracy in AF detection compared to other machine-learning approaches.
Lueken Markus, et al. [38]	Germany	Algorithm validation study	Validation of an AI-based algorithm for detecting AF in older patients using wearable devices for continuous monitoring.	Sensitivity: 92%, Specificity: 88%, with a significant improvement in AF detection time.	AI models in wearable devices can enhance continuous monitoring and reduce diagnostic delay in older patients.
Isaksen et al. [20]	Norway	Retrospective validation study	AI-based algorithm to detect AF in patients with multiple comorbidities using 12-lead ECG data.	Achieved 89% sensitivity and 90% specificity in detecting AF in complex cases.	AI can improve AF detection in patients with multiple comorbidities, supporting earlier interventions and better outcomes.

Legends: AI: Artificial Intelligence; AF: Atrial Fibrillation; AUC: Area Under the Curve; CNN: Conventional Neural Networks; ECG: Electrocardiogram; PPG: Photoplethysmography; NPV: Non-Pulmonary Vein; NSR: Normal Sinus Rhythm; SR: Sinus Rhythm; XAI: Explanable AI.

## Data Availability

Data available on request from the authors.
